# Temperature extremes and autoimmune diseases

**DOI:** 10.1007/s00281-026-01089-6

**Published:** 2026-10-13

**Authors:** Katharina Robichon, Rebecca Grainger

**Affiliations:** 1 Department of Pathology and Molecular Medicine, University of Otago Wellington, Wellington, New Zealand; 2 Department of Medicine, University of Otago Wellington, 23a Mein St Newtown Wellington South, Wellington, PO Box 7343, 6242 New Zealand; 3 Wellington Regional Rheumatology Unit, Hutt Hospital, Health New Zealand Capital Coast and Hutt Valley Health, Wellington, New Zealand

**Keywords:** Climate change, Temperature, Autoimmune diseases, Environmental Exposure

## Abstract

Temperature extremes linked to climate change are becoming more frequent and more intense. This has significant implications for human health, including conditions driven by immune dysregulation. There is growing evidence that external thermal stress, particularly heat, may influence both the development and clinical course of several autoimmune conditions. Heat and cold can alter immune cell function through effects on cell signalling, cytokine production, barrier integrity and stress response within tissues. These mechanisms provide a strong biological basis for the observed associations between seasonal or regional temperature patterns and rates of autoimmune disease onset, symptoms, or flare frequency. Temperature extremes present considerable specific health risk to people with autoimmune disease with examples including heat waves worsening fatigue and neurological symptoms in people with multiple sclerosis and cold spells triggering blood vessel constriction and extremity tissue necrosis in systemic sclerosis. As climate instability increases, people with autoimmune disease will face higher clinical risk, due to disease-related factors and increased risks from cardiovascular related morbidity and mortality and concomitant demographic and socio-economic factors. This highlights the need for climate-aware healthcare, patient education, and research that addresses the interface of environmental factors with immune and clinical outcomes.

## Introduction

Climate change is an existential threat to humanity that is of our own doing and can no longer be ignored. Human activity-generated climate change is contributing to temperature extremes (TE) that are becoming increasingly frequent, lasting longer, and extending geographically. The impacts of TE on human health are varied, large, and concerning, having impact on morbidity and mortality [1–4]. While these impacts on general health and health system have been addressed in detail elsewhere, the impact of TE on the human immune system and related diseases have not received much focus.

**Fig. 1:**
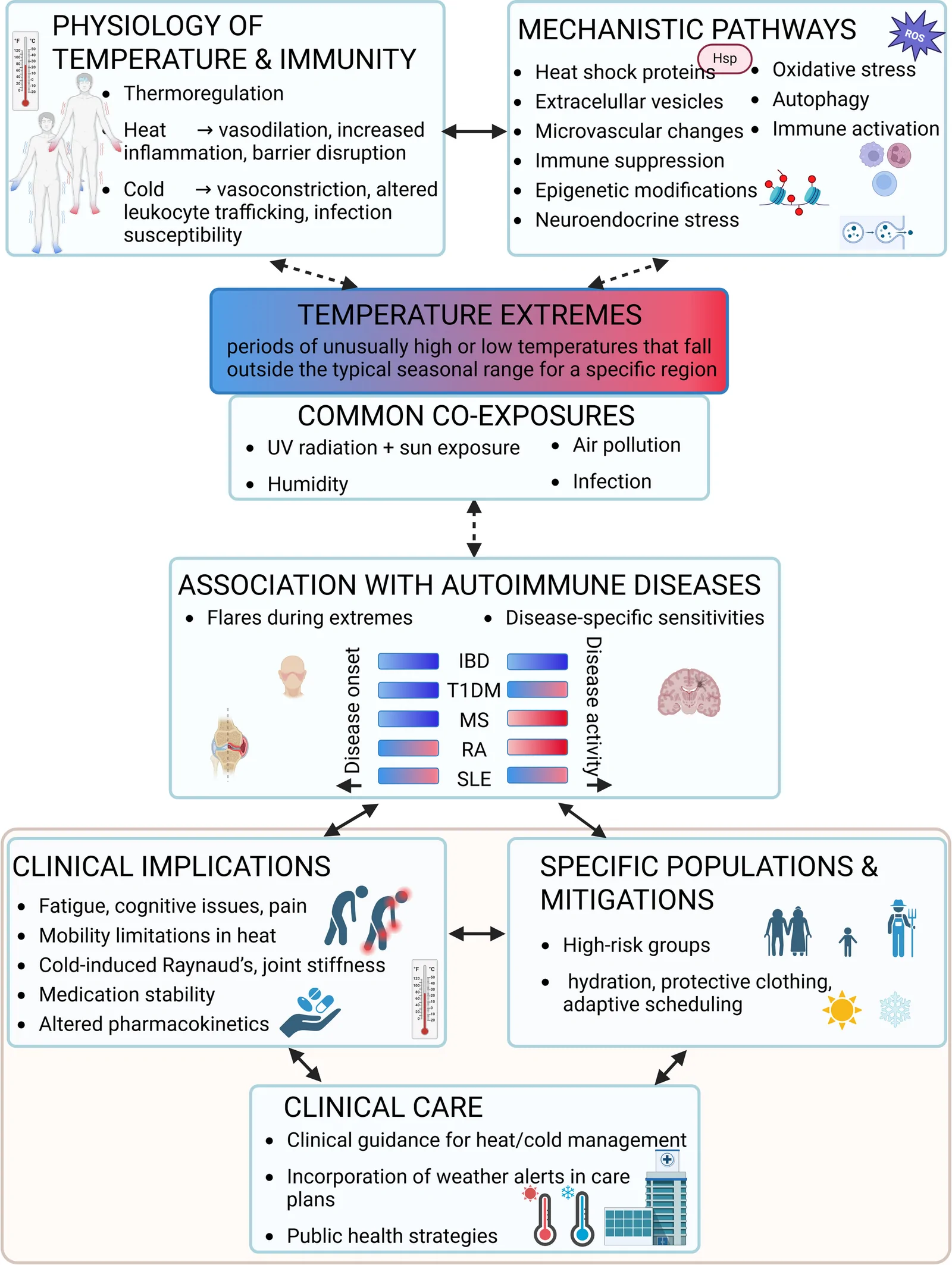
Model illustrating how temperature extremes influence autoimmune disease activity and outcomes. Temperature extremes—heatwaves, cold spells, and rapid fluctuations—affect the immune system through core thermoregulatory pathways and temperature-dependent changes in immune cell function. Epidemiologic data link these exposures to increased autoimmune disease flares and symptom severity. Mechanistic pathways include heat shock responses, oxidative stress, vascular changes, and infection-related effects. These contribute to a range of clinical consequences, particularly in vulnerable populations. Resulting implications for clinical care and public health underscore the need for mitigation strategies and highlight key knowledge gaps requiring future research. Solid arrows indicate direct influence, dashed arrows indicate indirect interaction. Coloured stripes in ‘Association with autoimmune disease’ box represents direction of association: blue bar – cold temperatures are associated with increased disease onset/disease activity; red bar – warm temperatures are associated with increased disease onset/disease activity, red/blue bar – inconclusive based on the current literature. *Figure created using BioRender. *(Abbreviations *HSP* heat shock proteins, *IBD* inflammatory bowel disease, *MS* Multiple sclerosis, *RA* rheumatoid arthritis, *ROS* reactive oxygen species, *SLE* systemic lupus erythematosus, *T1DM* Type one diabetes mellitus)

In this review, we aim to provide a comprehensive overview of the implications of climate change-related TE and autoimmune disease, with a focus on rheumatic disease and neurological disease (Fig. 1). We will consider the physiology of temperature and the immune system and discuss how TE could be anticipated to influence the immune system. We will outline the emerging epidemiology data linking TE to autoimmune disease and discuss postulated mechanistic pathways. Particular populations of interest and vulnerable groups will be considered, and clinical and public health implications discussed, including steps to mitigate negative impacts on human health. Finally, we will highlight knowledge gaps and suggest priorities for future research.

Before focusing on TE and autoimmune disease, we should note that humans have the capacity for short-term and adaptive thermoregulation using behavioural and autonomic mechanisms to maintain core body temperature at 37°C [5]. As such, humans live successfully in a wide variety of climatic conditions, from the Arctic to the Sahara. Therefore TE are considered in the context of the local climatic ranges. Definitions of TE have been proposed by the Intergovernmental Panel on Climate Change (IPCC) [6] and the World Meteorological Society (WMO) [7]. (Table 1) Extremes of both cold and heat can cause excess mortality, with an excess of deaths particularly amongst people over 65 years of age [8]. An understanding of the physiology of temperature control in humans and the influence on the immune system underpins understanding of potential impacts of TE on autoimmune disease.

**Table 1:** Definitions of temperature extremes from Intergovernmental Panel on Climate Change [6] and The World Meteorological Society [7]

Event	Intergovernmental Panel on Climate Change (IPCC)	The World Meteorological Society (WMO)
Extreme weather event (which includes temperature)	A weather event that is as rare or rarer than the 10th or 90th percentile of a probability density function estimated from observations. By definition the characteristics of what is called extreme weather may vary from place to place in an absolute sense.	Monitoring and describing of extreme events consider its intensity, temporal and spatial characteristics, with the definition being impact-independent, with the aim of developing a system that can report consistently on the nature, occurrence and evolution of the event. Event will be described narratively with relation to local conditions and evolution of the event.
Heat wave	A period of abnormally hot weather, often defined with reference to a relative temperature threshold, above 90th centile lasting from two days to months.	Narratively defined by National Meteorological and Hydrological Services according to local conditions. Working definition include periods of unusually hot and dry or hot and humid weather that have a subtle onset and cessation, a duration of at least two to three days and a discernible impact on human activities
Cold spell	Not specifically defined but could be interpreted as a period of abnormally cold weather below 10th centile lasting from two days to months.	Narratively defined by National Meteorological and Hydrological Services according to local conditions.

## Physiology of temperature regulation and the immune system

Temperature changes and immune system function are closely linked with influence at multiple levels. To maintain thermal homeostasis, the physiological processes of the nervous, endocrine, vascular systems are co-ordinated, alongside a variety of heat reducing or mitigating behaviours [5]. Peripheral immune sites, like the skin, are directly exposed to the changes in environmental temperature but also exposed to ultraviolet (UV) radiation. Central immune compartments, including circulating blood and the lymphoid tissue, are mainly influenced by core temperature. Autonomic mechanisms including vasodilation and sweating in response to heat increase the core temperatures, and peripheral vasoconstriction and shivering in response to cool temperatures help cool core body temperatures. These differences shape how various parts of the immune system response to thermal challenges.

The two arms of immune system, the innate and the adaptive systems, respond differently to thermal changes. The innate immune system provides the body with immediate and non-specific defences against pathogens through barriers like skin and innate immune cells such as neutrophils, macrophages, and dendritic cells. On contact with foreign antigens, the adaptive immune system is slower in response but highly specific. It relies on the activation of T and B lymphocytes by innate immune cells. These lymphocytes recognize antigens and generate immunological memory, enabling a stronger and faster response upon future exposure. The differences in the way the innate and adaptive immune systems respond to heat and cold arise from the unique cellular and molecular mechanisms that characterize each arm of the immune system [9].

Heat stress can lead to the cellular upregulation and release of heat shock proteins (HSPs), which act as a danger signal and modulate the responses of macrophages and dendritic cells. Elevated HSP levels have often been associated with disease activity or progression [10, 11]. The HSPs-associated induction of oxidative stress and an increase in the production of pro-inflammatory cytokines could promote a change in the adaptive immune response, especially in the balance between T helper 1 and T helper 17 lymphocyte differentiation, two specific T cell subsets highly associated with autoimmune diseases. However, cold stress downregulates general cytokine production resulting in a suppression of the innate immune response leading to reduced phagocytosis and dendritic cells activation [12]. This results in impaired T lymphocyte response, alters immune cell trafficking, and may change regular T and B lymphocyte activity [13]. While no direct research of high TE has been conducted, examining effects of high fevers has shown that elevated temperatures directly alter regular T cells leading to them being less effective. Murine CD4 T cells were cultured at homeostatic 37°C or physiological fever-range 39°C temperatures for 3–4 days and induced regular T cells were less effective at suppressing effector CD8 T cell proliferation, potentially due to an altered metabolism and upregulation of Glut1 [14]. In comparison, 33°C induced regular T cells were better at suppressing effector T cell proliferation in vitro than iTregs induced at 37°C, as evidenced from the decreased proliferation of activated CD4 + effector T cells [15, 16]. In patients with active rheumatoid arthritis and systemic lupus erythematosus, a significant decline in peripheral Bregs indicates an impaired capacity to suppress the inflammatory response, mediated by the provision of IL-10 and by direct contact with CD4 + T cells [17, 18].

Moreover, exposure to heat or cold stress not only affects immune cell function in the short term but can also trigger epigenetic and transcriptomic reprogramming, including Histone modification and DNA methylation, shaping future immune cells response [19]. A short summary of the thermal influence on the immune system is provided in Table 2. Although these thermal stress-related immune system changes are well described in animal and cell models, the direct link to the progression or exacerbation of human autoimmune diseases requires further research.

**Table 2:** Thermal influence on innate and adaptive immune system. Arrows up – upregulation of immune cell function, Arrows down – down regulation of immune cell function

Thermal Influence	Innate Link	Bridge	Adaptive Outcome
Fever/Heat	↑ Cytokines, HSPs, dendritic cell activation [11, 20, 21]	↑ Antigen presentation	↑ T lymphocyte response, possible autoimmunity
Cold	↓ Cytokines [12] ↑ anti-inflammatory macrophages	↓ dendritic cell activation [12]	↓ T lymphocyte response [13] ↑ Treg activity [16]

## Association of temperature extremes and autoimmune disease

Relationships between climate and autoimmune disease epidemiology at a global level were recently considered using disease incidence data from the Global Burden of Disease study (2019) and ambient climate data from the World Bank Climate change knowledge portal [20]. There was a consistent inverse linear association between temperature and aged-standardised rates of five autoimmune diseases with the relationship strongest for Type 1 diabetes mellitus (T1DM) (Adjusted R^2^ 0.478, *p* < 0.0001) and weakest for rheumatoid arthritis (RA) (Adjusted R^2^ 0.179, *p* < 0.0001). These data may suggest that overall rates of autoimmune disease may decline with increasing global temperatures. The authors also undertook a systematic literature review with a substantive literature supporting north-south gradient differences in onset and/or prevalence for T1DM, and inflammatory bowel disease (IBD) with higher rates in northern (colder) regions. Similarly, the incidence of multiple sclerosis (MS) is higher at higher latitudes in the northern hemisphere [21] and MS severity is also increased further north [22]. One proposed mechanism is lower Vitamin D levels, due to reduced sunlight UV exposure, in people living in in cooler (northern) regions with Vitamin D being an immune modulator. Only four studies focusing on RA were identified, with two reporting higher risk of onset in colder regions of the USA, and two reporting no association of RA rates with geographic variations, although these studies were from countries with less climatic variation (Sweden and Sardina).

These observations add to the longstanding interest in the effects of weather and latitude on autoimmune disease, particularly for the effects of weather on symptoms of RA [23]. Generally studies to date have been conflicting and inconclusive with major shortcomings being short duration, lack of use of standardised outcomes measures, and heterogenous descriptors for weather conditions. More recent literature exploring environmental impacts on autoimmune disease activity and outcomes has recently been summarised [24] with most literature focused on air pollutants and only a handful of studies relevant to TE. A four-year study in a city in eastern China with a subtropical, mild climate found that diurnal temperature ranges at the 95th percentile were associated with increased rates of hospital admissions for people with RA, with the highest risk occurring after several cumulative days of large temperature fluctuations [25]. This population-based study did not ascertain reasons for admission, and overall rates of admission were low. A small retrospective study from Morocco found no consistent correlation of RA disease activity parameters with climatic conditions, although the included participants had well controlled disease with most in remission [26]. Studies examining relationship between systemic lupus erythematosus (SLE) activity and temperatures analysed by month [27] and season [28, 29] have heterogenous findings, thus, the effects of temperature extremes on SLE onset or activity remain unclear. High environmental temperatures have been associated with increased rates of admission with gout in a population-based study in China [30] and associated with 40% increased rates of gout flares in a case-crossover study in the USA [31] with the highest rate of gout flare seen in both hot and humid conditions. This has mechanistic plausibility where cooling sweat is less effective with high humidity, worsening dehydration leading to acute gout.

Multiple sclerosis is a prototypic autoimmune neurological disease with symptoms reported to be affected by temperature. Single-centre studies have found different associations between meteorological variables and MS relapses, where some found no association [32, 33] and others found that general relapses were more frequent in the summer months [34, 35]. It is interesting to note that the single-centre studies that did not find an association seemed to be positioned closer to the equator, where temperature changes are not that extreme and the level of MS incidence might be lower. A primary survey study by Christogianni et al. showed that hot days and high humidity are the main triggers for heat sensitivity with 66% reporting fatigue and weakness as main symptoms [36].

In summary, the data on temperature and autoimmune disease epidemiology and disease activity are limited in number, often single location, and tend to focus on ambient temperatures. Understanding the mechanistic pathways by which thermal stress may influence autoimmune disease may provide biologically based hypotheses to focus the design of future studies of TE and autoimmune disease epidemiology or clinical studies.

## Mechanistic pathways

The mechanistic pathways underlying immune responses to thermal stress specifically as related to autoimmune diseases are complex and multifactorial. Understanding the cellular and molecular mechanisms by which thermal stress modulates the immune system is critical for elucidating how environmental temperature fluctuations influence autoimmune disease.

### Heat related pathways

Based on existing available literature, the link between the induction of heat-shock proteins (HSPs) and the anti-HSP autoantibodies in the development of autoimmune diseases seems relevant however precise links remain unclear. Autoantibodies are produced by the adaptive immune system, specifically by B cells, the precursor to plasma cells. Autoantibody production reflects a breakdown in immune tolerance, a key regulatory function of adaptive immunity. The occurrence of autoantibodies is a common feature of autoimmune diseases. HSPs are a family of proteins produced by all cells in response to external stress. They act as molecular chaperons to monitor if proteins are folded correctly and mark damaged proteins for degradation. HSPs are regulated through heat-shock factor 1 (HSF-1). In the steady state HSF-1 is bound to HSP70 and HSP90. If heat stress is encountered it leads to the dissociation of HSF-1 and its translocation into the nucleus and induction of HSPs expression. In addition, HSPs can be released into extracellular space and be presented to T cells by major histocompatibility complex (MHC) molecules [10]. This interaction with the immune system might stimulate the humoral immune response leading to the generation of anti-HSP autoantibodies which can both be protective and pathogenic [10]. Numerous studies, reviewed by Tukaj et al. 2024, have shown that elevated levels of anti-HSP autoantibodies can be detected in patients suffering from numerous different autoimmune diseases, including SLE, RA, juvenile idiopathic arthritis, myasthenia gravis, psoriasis, coeliac disease but not MS [10]. In RA, increased levels of pro-inflammatory cytokines interleukin-6 (IL-6) or interferon-gamma have been positively correlated with higher titres of autoantibodies directed against HSP40 or HSP90 respectively [37, 38]. While a detailed review of the different HSPs in autoimmune diseases including structure and location and precise immunomodulatory effects is available [39], the pathological role of anti-HSP autoantibodies is not fully understood.

In addition to HSP facilitated autoantibody production, the disruption of protein folding due to heat can lead to the formation of neoantigens which can undergo post-translational modification. These can trigger autoimmune diseases by exposing novel epitopes to the immune system presented by cells as “non-self” leading to the expansion of autoreactive T cells [40]. Nonetheless, neoantigen formation in autoimmune diseases has been linked not only to heat but also to a range of environmental factors, including smoking, ultraviolet light exposure, and infectious agents, making it difficult to isolate the specific contribution of heat [41–43].

Heat-induced stress can further lead to endothelial injury and induced endothelial barrier dysfunction either through direct thermal cytotoxic effects or through secondary-induced systemic inflammatory response syndrome. Damage and activation of endothelial cells lead to the activation and increase of adhesion molecules, such as intercellular adhesion molecular-1 (ICAM-1) and vascular cell adhesion molecule-1 (VCAM-1). In addition, downregulation of junctional proteins leads to increased leukocyte-endothelial adhesion, increased vascular permeability, and subsequent inflammatory infiltration [44]. MS is characterized by increased permeability of the blood-brain barrier consisting of endothelial cells surrounded by astrocytes, pericytes, and smooth muscle cells. Thus, heat stress-induced endothelial hyper-permeability might increase inflammatory infiltration into the central nervous system (CNS) which could worsens MS symptoms [45]. Furthermore, endothelial damage leads to the production of pro-inflammatory cytokines and HSPs which induces nitric oxide synthase (iNOS) and release of nitric oxide (NO) leading to increased vascular dysfunction and further adhesion molecules upregulation [44]. Heat stress can also accelerate autoimmune tissue damage by altering extra-cellular vesicles (EV) cargo, boosting pro-inflammatory pathways, and triggering immune cell activation. Temperature elevations affect the actual content packaging of EVs, while RNA content is reduced with increased heat, proteins associated with metabolism and protein synthesis are increased [46]. Furthermore, EVs released by heat-stressed tissue cells are sufficient to cause further tissue damage to healthy bystander tissue cells leading to higher levels of DNA damage and apoptosis [47].

TE significantly alter the balance between pro-inflammatory and pro-resolving mediators in autoimmune diseases and an altered specialized pro-resolving mediator’s profile has been observed in different rheumatic diseases, including SLE and RA [48]. Moderately elevated temperatures, like mild fevers, can enhance immune function by acting synergistically with specialized pro-resolving mediators. They upregulate SPM receptors expression on immune cells and can promote clearance of cellular debris and apoptotic cells. However, extreme heat can destabilize these lipid mediators [49].

The constant activation and prolonged proliferation of immune cells due to pro-inflammatory cytokines, tissue damage, and other signals can lead to stress-related exhaustion of immune cells, particularly T cells, leading to dysfunction. This could lead to autoimmune flares [50]. However, T cell exhaustion in autoimmune disease may also have the beneficial effect of attenuating disease activity and preventing relapse as seen in SLE [51].

### Cold-related pathways

While heat related pathways in autoimmune diseases often involve enhanced cytokine production and cellular responses that promote inflammation, cold-related pathways trigger distinct yet complementary mechanisms of immune activation.

Cryoglobulins are immunoglobulins that precipitate in cold temperatures and can occur in autoimmune diseases. Mixed cryoglobulins from the cold-precipitable rheumatoid factors (RFs) are frequently detected in Sjogren’s disease and RA [52]. Cryoglobulin precipitation (cold-induced immune complexes) triggers an inflammatory cascade that includes complement activation, which can cause vasculitis and organ damage. Cryoglobulinaemic vasculitis is an infrequent disease manifestation of RA, Sjogren’s disease, and SLE [53] but could become more frequent with increased exposures to extreme cold.

### Temperature change pathways

Several mechanistic pathways are induced by either cold- or heat-related exposure. TE can cause mitochondrial dysfunction through damage to the electron transport chain and inflammation. Oxidative stress and mitochondrial dysfunction are closely linked in immune system dysregulation and several autoimmune diseases. Malfunctioning mitochondria within crucial immune cells may disrupt the establishment of regulatory networks [54]. Jing et al. (2023) reviewed how the hypoxic microenvironment of RA joints can cause reactive oxygen species (ROS) accumulation and mitochondrial damage leading to upregulated expression of several inflammatory pathways and dysfunction of immune cells [55]. This change in metabolic activity could be causing flares in RA where RA T-cells have a defect in glycolytic flux [56]. Similarly, mitochondrial oxidative stress is a potential driver in SLE where O_2_ consumption was found to be increased in peripheral blood relative of people with SLE compared to control subjects [57]. The oxidative damage through ROS production can also have a negative effect on the intestinal mucosal barrier, leading to increased permeability and systemic inflammation which has been reported as a leaky gut syndrome in RA, SLE, T1DM, IBD [58]. Rio et al. (2024) reviewed the impact of climate change on immunity and gut microbiota and noted the TE to influence gut microbiota with potential to increase the risk of developing autoimmune and allergic chronic inflammatory diseases [59]. In addition, mitochondrial stress and ROS production can also activate autophagy and both cold exposure and heat stress can upregulate autophagy, playing a dual role in autoimmune diseases, like SLE, MS and RA. In people with MS, upregulation of the autophagic ATG5 protein has been described in T cells infiltrating inflammatory sites [60], this might lead to degradation of pro-caspases in T cells, antagonizing apoptosis and implicating autophagy in the survival of autoreactive T cells [61]. In RA, the insufficient autophagic fluxing contributes to immune abnormalities by switching to lower metabolic activity of naïve CD4 T cells [62]. Heat changes effect on isolated PBMCs have shown that at 37 °C an increased autophagic activity was demonstrated which increases with increasing temperatures (up to 41 °C), while there was no observation in increased apoptosis under the tested temperature condition [63]. In addition, also cold exposure increased autophagic and heat shock protein activity but also increased apoptosis with decreasing temperature [64].

In addition, increased sun exposure can also compromise the barrier function of the skin which might exacerbate cutaneous lupus, psoriasis, or atopic dermatitis [65]. Furthermore, the autonomic nervous system (ANS) uses neurotransmitters to directly influence immune cells. Extreme temperature changes can disrupt the ANS function leading to chronic inflammation, immune dysregulation, and exacerbating symptoms in people with autoimmune conditions like RA and SLE [66].

## Clinical implications for individual patients with autoimmune diseases

Human core body temperature is tightly regulated to typically maintain within 1°C range. The key regulatory mechanisms are behavioural, for example humans will move environments and/or remove or add clothing to maintain thermal comfort. Physiological mechanism to maintain core body temperature include sweating (to cool) and arteriovenous shunt vasoconstriction and shivering (to maintain temperature) [5]. Exposure to TE that are beyond thermoregulatory capacity has direct impacts on disease manifestations for individuals with autoimmune diseases, both negative and positive. Although this review focuses on rheumatic and neurological autoimmune disease, clinically important interactions between TE and other autoimmune and immune-mediated diseases have also been described. These interactions may occur through direct effects of thermal stress on immune-mediated disease activity or through precipitation of disease-specific complications resulting from impaired physiological adaptation to heat or cold, with representative examples provided in Table 3. These examples illustrate that exposure to TE can have negative or positive effects on a disease or its manifestations. For example, exposure to cold in autoimmune rheumatic diseases with vascular involvement, such as systemic sclerosis, can threaten tissue integrity with ischaemia secondary to dysregulation of normal vasoconstriction. In contrast, cold exposure can have benefits in MS while exposure to extreme heat worsens symptoms of MS. Furthermore, TE are seldom experienced without exposure to other environmental exposures, such as sunlight. Sun exposure can directly trigger skin manifestations of SLE and worsen internal organ involvement due to UV light induced activation of skin immune processes. We will explore how temperature variations and TE and associated environmental exposures may be associated with potential or actual clinical implications in detail for two rheumatic diseases, systemic sclerosis and SLE, and in MS.

**Table 3:** Representative examples of the clinical consequences of temperature extremes across autoimmune and immune-mediated diseases. Section A summarises diseases in which temperature extremes are associated with changes in immune-mediated disease activity or clinical manifestations. Section B summarises diseases in which temperature extremes primarily precipitate complications through disease-related physiological vulnerability rather than direct effects on autoimmune activity. Examples are illustrative and not exhaustive

Disease	Temperature extreme	Principal clinical consequence	Primary mechanism	Key references
A. Temperature extremes influencing autoimmune disease activity or manifestations				
Multiple sclerosis	Heat waves	Uhthoff phenomenon, fatigue and transient neurological deterioration	Temperature-dependent conduction block in demyelinated axons; increased blood–brain barrier permeability	31, 65–69
Systemic sclerosis	Cold spells	Raynaud phenomenon, digital ischaemia, ulceration and tissue necrosis	Cold-induced vasoconstriction superimposed on autoimmune microangiopathy	53–58
Systemic lupus erythematosus	Heat and cold extremes (often with concomitant UV exposure)	Increased disease activity, cutaneous flares and vascular complications	UV-induced immune activation, endothelial dysfunction and vascular injury	23, 59–62
Rheumatoid arthritis	Large diurnal temperature variation and cold exposure	Increased symptoms and hospital admissions	Altered inflammatory and metabolic pathways	20, 21
Cryoglobulinaemic vasculitis	Cold spells	Vasculitic flares, purpura, digital ischaemia and tissue injury	Cold-induced cryoglobulin precipitation with complement activation	43, 44
Cold agglutinin disease*	Cold spells	Haemolysis, acrocyanosis and circulatory symptoms	Cold-dependent IgM binding to erythrocytes with complement activation	
B. Temperature extremes precipitating complications due to disease-related physiological vulnerability				
Grave’s disease	Heat waves	Worsening heat intolerance and increased risk of thyrotoxic decompensation (including thyroid storm with concurrent physiological stress)	Autoimmune hyperthyroidism causes excess metabolic heat production and impaired heat dissipation	
Autoimmune hypothyroidism (Hashimoto disease)	Cold spells	Myxoedema coma in susceptible individuals	Autoimmune hypothyroidism impairs thermogenesis and adaptive responses to cold	
Autoimmune adrenal insufficiency (Addison disease)	Heat waves	Adrenal crisis precipitated by dehydration and physiological stress	Impaired cortisol and aldosterone responses limit adaptation to heat stress and volume depletion	
Myasthenia gravis	Heat waves	Worsening muscle weakness and fatigability, occasionally precipitating myasthenic crisis	Elevated temperature impairs neuromuscular transmission at the already compromised neuromuscular junction	

*Cold agglutinin disease is included as a prototypical example of temperature-dependent immune pathology, although it is often considered separately from the systemic autoimmune diseases discussed in this review

Dysregulation of peripheral vascular thermoregulating mechanisms occurs in Raynaud’s phenomenon (RP). A key feature of RP is an episodic painful vasospastic phenomenon where an exaggerated vasoconstriction causes digital ischemia (white phase) and can have a cyanotic (blue) and reperfusion (red) phases [67]. RP affects 3–5% of the population with the majority being primary RP with no underlying systemic disease. Secondary RP occurs in several autoimmune rheumatic diseases including systemic sclerosis, SLE, RA, and anti-synthetase syndrome.

### Systemic sclerosis

Systemic sclerosis, also known as scleroderma, is a rare autoimmune disease characterised by skin and (variable) internal organ fibrosis with universal micro vasculopathy [68]. Due to the near ubiquitous feature of Raynaud’s phenomenon, and co-existent microvascular disease, people with systemic sclerosis can develop digital ulceration, and have potential for digital necrosis. The incidence of systemic sclerosis vascular complications is significant, with complications requiring admission and/or significant medical treatment occurring in almost 18% of a scleroderma patient reviewed in a large UK specialist centre [69].

A recent Australian national study in a large systemic sclerosis cohort showed that patients reporting worsening RP had been exposed to lower mean minimum and maximum temperatures, measured at the governmental weather monitoring station nearest their residential address in the preceding month, compared to those not reporting worsening RP [70]. Furthermore, people with systemic sclerosis had decreased odds of reporting worsening RP with higher mean maximum temperatures (odds ratio [OR] 0.91,95% confidence interval [95% CI] 0.90–0.92, *P* < 0.001) and lower mean daily evaporation (OR 0.77, 95% CI 0.73–0.81, *P* < 0.001), but increased odds with high relative humidity (OR 1.05, 95% CI 1.04–1.05, *P* < 0.e001). This gives an estimated 9% decreased risk of reporting worsening RP for each increase in mean maximum temperature of 1 °C. The strong influence of weather on RP in systemic sclerosis was also demonstrated in a ten-year longitudinal study in over 2000 people with systemic sclerosis. It examined the relationship between RP severity scores in the preceding week and local temperatures, as measured by government weather bureaus [71]. The worst RP scores were observed at − 25 °C with minimal improvement as temperatures increased from − 15 °C to + 5 °C. RP scores then improved through to + 25 °C, only to worsening again at higher temperatures. These data confirmed the worsening of RP frequency with both cold and hot TE, with the increase in RP at higher temperatures proposed to be due to exposures to air-conditioned interior environments in hotter ambient weather, a behavioural adaption to high TE. While it has been suggested that the incidence of primary RP may decrease with predicted global warming [72], this may not be realized due to increased use of air-conditioned environments. Overall, these data suggest that exposure to TE poses significant health risks for people with systemic sclerosis. Cold extremes have a clear association with increased risk of RP, poorer health-related quality of life, and a higher likelihood of digital ischaemia [70]. Hot extremes may also increase the risk of RP, potentially due to greater reliance on air conditioning.

### Systemic lupus erythematosus

Several studies have explored the impacts of TE on SLE disease activity, with both warmer seasons and lower temperatures associated with adverse outcomes. A recent study in China examined hospital admissions and re-admissions for SLE over a 10-year period and found that low ambient temperatures increased SLE hospital re-admission with an odds ratio of 2.53 (for 3.5 °C vs. 21 °C) with an effect lasting for two weeks [73]. There was no effect of temperature on (first) admissions. A detailed analysis of the Hopkins lupus cohort showed that flares of skin and joint manifestations were more frequent in spring and summer months, while other organs had different activity patterns [28]. Mechanisms underlying these patterns could include UV exposure, HSP induction and other danger signals in skin detected by immune cells. A longitudinal study of people with SLE in Hong Kong reported increased renal lupus activity in winter months and the rate of SLE flares showed a U-shaped curve with higher rates at the extremes of 16 °C and 30 °C, with a similar pattern seen for extremes of ultraviolet light exposure [74].

While less common manifestations in SLE, Raynaud’s phenomenon, lupus vasculitis and lupus chilblains are all direct pathologies of blood vessels. In addition, the frequent coexistence of atherosclerotic vascular disease and anti-phospholipid syndrome further increases the risk for skin and digital ischaemia in cold conditions in people with lupus [75]. Dramatic case reports of gangrene in women with lupus after short-lived exposure to extreme cold highlight this risk [76].

### Multiple sclerosis

MS is an autoimmune disease characterized by continuous infiltration of autoreactive T cells and other inflammatory immune cells from the periphery into the CNS. The pathology of MS is characterised by damage to the myelin sheaths surrounding nerve axons, which causes demyelination, leading to sensory dysfunction and physical and visual impairments [77]. MS can be affected by both cold and heat stress.

Murine models have provided data to suggest cold exposure may influence MS onset and severity. Spiljar et al. demonstrated that exposing mice to cold temperatures either before induction or during the animal model of MS, reduced disease severity and reduced immune cell activation in the blood and the CNS, suggesting that cold exposure decreases immune cell pathogenicity [78]. One proposed explanation is that the cold exposure provokes a high metabolic cost shift by increasing heat production in brown adipose tissue. This energetic demand diverts energy and resources from other systems, including the immune system. Reducing the energy source for immune cells may reduce the activity of immune and inflammatory cells.

In contrast the symptoms of MS can be worse in the heat, with Uhthoff’s phenomenon occurring in 60–80% of people living with MS (PwMS) [79]. The Uhthoff’s phenomenon is characterised by short episodes (minutes to hours) of blurred vision that mimic optic neuritis [80]. While mechanisms are poorly understood, it is proposed that heat may slow or block nerve impulses in demyelinated neurons and lesions in thalamus or cortical regions in the brain may cause thermoregulation dysfunction. Carefully history taking to identify potential heat-related, infection, menstrual cycle or stress-related precipitants is critical to differentiate from optic neuritis to avoid unnecessary exposure to glucocorticoids [80]. It has been shown that MS attacks are more frequent in the warmest and coldest months of the year [81] but that PwMS experience more fatigue in summer months compared to winter, which might increase the risk of relapse [82]. The increased relapse rate in association with heat might also be due to increased permeability of the blood-brain barrier allowing more autoreactive immune cells to enter the CNS [83].

As temperatures rise, people may spend more time indoors, particularly in regions becoming too hot which limits the exposure to sunlight. Vitamin D deficiency is a significant environmental risk factor for MS. Thus, the overall disease burden could shift, with a potential increase in MS prevalence in areas affected by altered sunlight exposure patterns.

## Specific populations and clinical implications

Exposure to TE requires behavioural and physical adaptations to avoid temperature-related health impact. Not all members of communities are able to make behavioural changes and/or have sufficient physiology adaptability to tolerate extreme heat or cold. People over 65 years of age, infants and younger children, pregnant people, and those with pre-existing illnesses or disabilities are at increased risk of heat-related illness [84] Socio-economic factors including such as lack of housing, inadequate finances to run air-conditioning, employment in outdoor work and residence in urban areas also increase risks of heat-related illness. These factors are also associated with increased incidence and prevalence of autoimmune rheumatic diseases such as SLE and RA [85]. RA prevalence is higher in older people, who are more susceptible to extremes of heat. Likewise, the prevalence of cardiovascular disease is higher in autoimmune rheumatic disease and pre-existing CVD is a major risk factor for mortality during heat waves [84, 86].

The accumulation of risk for poor health outcomes in combination with exposure to extreme heat suggests that those providing medical care for people with autoimmune disease should take personal steps to reduce their climate impact and advocate for change at service and country level [87, 88]. Furthermore, more climate-sensitive health counselling is rapidly becoming an expected part of clinical care [89]. Domains that are important in climate-sensitive health counselling include health impacts and mitigations, healthy and sustainable lifestyles, and climate action. Education about the risk of heat and heat waves, mitigation strategies and early health seeking behaviour should all be communicated to patients, particularly those with additional vulnerabilities [84]. Additional advice about medications is warranted. At high temperatures sweating leads to risk of dehydration. Patients should be advised to withhold potentially nephrotoxic agents such as non-steroidal anti-inflammatory drugs(NSAIDs), and medications that can rapidly accumulate and cause toxicity with reduced renal secretion, such as methotrexate. Patients should be reminded that biologic medications for rheumatic disease or MS disease need to be stored in climate-controlled conditions.

## Knowledge gaps and future research

Currently, there is a lack of longitudinal mechanistic studies exploring how thermal stress and changes in temperatures may contribute to the initiation and progression of autoimmune diseases. These longitudinal cohort studies should be carried out across diverse climate zones to increase the understanding of these relationships. Individual‘s heat exposure is dependent on time spent outdoors, travel, clothing and local environment like access to air-conditioning. Integrating data from wearable sensors and potential digital biomarkers, while expensive, could provide insights into individual physiological responses to temperature changes. Digital biomarkers could include monitoring the autonomic response to thermal stress by measuring the heart rate or analysing the sweat rate and composition. Monitoring these additional biomarkers could help identify early physiological changes before clinical symptoms appear. It is important to consider interactions or synergistic effects with other environmental factors such as pollution, UV radiation, and infections. Thus, it is necessary to consider the interconnection between social and environmental risk factors.

Further research should also consider the impact of TE on medication stability and pharmacokinetics. Heat or cold extremes could alter the absorption, distribution, metabolism and secretion of drugs potentially reducing its efficacy. These effects might be particularly relevant for patients that rely on temperature-sensitive medications, like immunosuppressants or vasodilators. These approaches could also open opportunities for precision medicine by identifying thermal sensitive phenotypes and tailoring interventions and treatments accordingly. The goal is to find and consider interventions that have multilevel benefits and focus on social risk factors, individual’s response to heat or cold and treatment efficacy. To implement these benefits, it is important to make changes on multiple levels, not only on the individual level but also on community level including the health care system and national level [87].

## Conclusions

TE are increasingly common and can influence immune function and features and clinical outcomes of autoimmune disease. There are biological plausibility, mechanistic evidence and emerging epidemiological and clinical data to indicate that both heat and cold stress can have an impact on autoimmune disease. Although current evidence remain heterogeneous and focused on single settings or diseases, the consistency of observations around vascular complications in systemic sclerosis, temporal variations in flares in SLE, and heat-induced worsening in MS suggest that TE are clinically significant exposures for patients with autoimmune diseases and therefore for health care systems.

Therefore, there is an urgent clinical imperative for routine care for people with autoimmune disease to consider environmental context, particularly for those with additional vulnerabilities due to age, comorbidity, or socioeconomic disadvantage. Climate-sensitive health counselling will need to become part of care. It should include personalised risk assessments, guidance on risk-mitigation strategies, advice on medication storage and handling, hydration and clear instruction on when to seek care advice.

The knowledge gaps also need to be addressed. These include mechanistic studies, longitudinal and cross-sectional cohort studies, and the efficacy and costs of interventions to manage TE. Future research should also determine whether distinct environmental endotypes exist within autoimmune diseases. Rather than assuming uniform susceptibility to temperature extremes, patients may differ substantially according to underlying vascular dysfunction, autonomic regulation, thermoregulatory capacity, immune activation pathways, comorbidity and socioeconomic circumstances. Identifying these environmentally vulnerable subgroups could support precision medicine approaches by enabling targeted counselling, monitoring and interventions during periods of extreme heat or cold. The impacts of TE should also be considered holistically in the wider setting of climate change and environmental degradation, considering impacts between TE, UV exposure, pollution, and social determinants of health. Increasing TE events will be part of climate change and the health impacts for people with autoimmune diseases must be addressed.

## Data Availability

This is a review article so there are no primary data able to be made available. Figure 1 was created using BioRender.

## References

[1] Bell ML, Gasparrini A, Benjamin GC (2024) Climate change, extreme heat, and health. N Engl J Med 390:1793–801. 10.1056/nejmra2210769 38749034

[2] Kazi DS, Katznelson E, Liu C-L et al (2024) Climate Change and Cardiovascular Health. JAMA Cardiol 9:748–757. 10.1001/jamacardio.2024.1321 PMC11366109 38865135

[3] Ige-Elegbede J, Powell J, Pilkington P (2024) A systematic review of the impacts of extreme heat on health and wellbeing in the United Kingdom. Cities Heal 8:432–446. 10.1080/23748834.2023.2283240

[4] Zhu Z, Ji B, Tian J et al (2025) Heat exposure and respiratory diseases health outcomes: an umbrella review. Sci Total Environ 970:179052. 10.1016/j.scitotenv.2025.179052 40056553

[5] Sessler DI (2016) Perioperative thermoregulation and heat balance. Lancet 387:2655–2664. 10.1016/s0140-6736(15)00981-2 26775126

[6] International Panel on Climate Change (2022) Annex I: glossary. Global Warming of 1.5°C: IPCC special report on impacts of global warming of 1.5°C above pre-industrial levels in context of strengthening response to climate change, sustainable development, and efforts to eradicate poverty. Cambridge University Press. https://www.ipcc.ch/sr15/

[7] World Meterological Organisation (2023) Guidelines on the definition and characterization of extreme weather and climate events. https://library.wmo.int/

[8] Ebi KL, Vanos J, Baldwin JW, Bell JE et al (2021) Extreme weather and climate change: population health and health system implications. Annu Rev Public Heal 42:293–315. 10.1146/annurev-publhealth-012420-105026 PMC9013542 33406378

[9] Balcerowska M, Kwaśnik P (2025) The multifaceted impact of stress on immune function. Mol Biol Rep 52:1008. 10.1007/s11033-025-11134-6 PMC12513959 41071399

[10] Tukaj S (2024) Dual role of autoantibodies to heat shock proteins in autoimmune diseases. Front Immunol 15:1421528. 10.3389/fimmu.2024.1421528 PMC11187000 38903496

[11] Tukaj S, Kaminski M (2019) Heat shock proteins in the therapy of autoimmune diseases: too simple to be true? Cell Stress Chaperones 24:475–479. 10.1007/s12192-019-01000-3 PMC6527538 31073900

[12] Hu G-Z, Yang S-J, Hu W-X et al (2016) Effect of cold stress on immunity in rats. Exp Ther Med 11:33–42. 10.3892/etm.2015.2854 PMC4726882 26889214

[13] Zieziulewicz TJ, Mondal TK, Gao D et al (2013) Stress-induced effects, which inhibit host defenses, alter leukocyte trafficking. Cell Stress Chaperones 18:279–291. 10.1007/s12192-012-0380-0 PMC3631090 23111563

[14] Heintzman DR, Sinard RC, Fisher EL et al (2024) Subset-specific mitochondrial stress and DNA damage shape T cell responses to fever and inflammation. Sci Immunol 9:eadp3475. 10.1126/sciimmunol.adp3475 PMC11607909 39303018

[15] Wilander BA, Rathmell JC (2025) Metabolic and stress response adaptations in T cells to fever and physiological heat. Trends Immunol 46:195–205. 10.1016/j.it.2025.01.007 PMC11922645 39984354

[16] Marek-Trzonkowska N, Piekarska K, Filipowicz N et al (2017) Mild hypothermia provides Treg stability. Sci Rep 7:11915. 10.1038/s41598-017-10151-1 PMC5607276 28931834

[17] Flores-Borja F, Bosma A, Ng D et al (2013) CD19 + CD24hiCD38hi B cells maintain regulatory T cells while limiting TH1 and TH17 differentiation. Sci Transl Med 5:173ra23. 10.1126/scitranslmed.3005407 23427243

[18] Blair PA, Noreña LY, Flores-Borja F et al (2010) CD19 + CD24hiCD38hi B Cells Exhibit Regulatory Capacity in Healthy Individuals but Are Functionally Impaired in Systemic Lupus Erythematosus Patients. Immunity 32:129–140. 10.1016/j.immuni.2009.11.009 20079667

[19] Casali C, Galgano L, Zannino L et al (2024) Impact of heat and cold shock on epigenetics and chromatin structure. Eur J Cell Biol 103:151373. 10.1016/j.ejcb.2023.151373 38016352

[20] Voskarides K, Philippou S, Hamam M et al (2025) Prevalence of autoimmune diseases is strongly associated with average annual temperatures: systematic review and linear regression analysis. BMC Rheumatol 9:86. 10.1186/s41927-025-00532-9 PMC12243313 40640896

[21] Simpson S, Blizzard L, Otahal P et al (2011) Latitude is significantly associated with the prevalence of multiple sclerosis: a meta-analysis. J Neurol Neurosurg Psychiatry 82:1132. 10.1136/jnnp.2011.240432 21478203

[22] Vitkova M, Diouf I, Malpas C et al (2022) Association of Latitude and Exposure to Ultraviolet B Radiation With Severity of Multiple Sclerosis. Neurology 98:e2401–e2412. 10.1212/wnl.0000000000200545 PMC9231838 35410900

[23] Patberg WR, Rasker JJ (2004) Weather effects in rheumatoid arthritis: from controversy to consensus. A review. J Rheumatol 31:1327–1334 15229951

[24] Katsumoto TR, Stolyar L, Dandeniya CL et al (2024) Impact of climate change on rheumatic diseases: A scoping review. J Clim Chang Heal 19:100338. 10.1016/j.joclim.2024.100338 PMC12851271 41647313

[25] Wang J, Yu L, Deng J et al (2022) Short-term effect of meteorological factors on the risk of rheumatoid arthritis hospital admissions: a distributed lag non-linear analysis in Hefei, China. Environ Res 207:112168. 10.1016/j.envres.2021.112168 34655606

[26] Azzouzi H, Ichchou L (2020) Seasonal and Weather Effects on Rheumatoid Arthritis: Myth or Reality? Pain Res Manag 2020:5763080. 10.1155/2020/5763080 PMC7492902 32963656

[27] Hua-Li Z, Shi-Chao X, De-Shen T et al (2011) Seasonal distribution of active systemic lupus erythematosus and its correlation with meteorological factors. Clinics 66:1009–1013. 10.1590/s1807-59322011000600015 PMC3129944 21808867

[28] Duarte-García A, Fang H, To CH et al (2012) Seasonal variation in the activity of systemic lupus erythematosus. J Rheumatol 39:1392–8. 10.3899/jrheum.111196 PMC3597113 22660806

[29] Chiche L, Jourde N, Ulmann C et al (2012) Seasonal variations of systemic lupus erythematosus flares in southern France. Eur J Intern Med 23:250–4. 10.1016/j.ejim.2011.12.006 22385883

[30] He Y-S, Wang G-H, Wu Z-D et al (2022) Association between non-optimal temperature and hospitalizations for gout in Anqing, China: a time-series analysis. Environ Sci Pollut Res 29:13797–13804. 10.1007/s11356-021-16580-w 34599442

[31] Neogi T, Chen C, Niu J et al (2014) Relation of temperature and humidity to the risk of recurrent gout attacks. Am J Epidemiol 180:372–7. 10.1093/aje/kwu147 PMC4184385 24993733

[32] Salvi F, Bartolomei I, Smolensky MH et al (2010) A seasonal periodicity in relapses of multiple sclerosis? A single-center, population-based, preliminary study conducted in Bologna, Italy. BMC Neurol 10:105. 10.1186/1471-2377-10-105 PMC2988761 21040535

[33] Fonseca AC, Costa J, Cordeiro C et al (2009) Influence of climatic factors in the incidence of multiple sclerosis relapses in a Portuguese population. Eur J Neurol 16:537–539. 10.1111/j.1468-1331.2008.02528.x 19187257

[34] Goodkin DE, Hertsgaard D (1989) Seasonal Variation of Multiple Sclerosis Exacerbations in North Dakota. Arch Neurol 46:1015–1018. 10.1001/archneur.1989.00520450085025 2775006

[35] Bamford CR, Sibley WA, Thies C (1983) Seasonal variation of multiple sclerosis exacerbations in Arizona. Neurology 33:697–697. 10.1212/wnl.33.6.697 6682514

[36] Christogianni A, O’Garro J, Bibb R et al (2022) Heat and cold sensitivity in multiple sclerosis: A patient-centred perspective on triggers, symptoms, and thermal resilience practices. Mult Scler Relat Disord 67:104075. 10.1016/j.msard.2022.104075 35963205

[37] Tukaj S, Kotlarz A, Jóźwik A et al (2010) Cytokines of the Th1 and Th2 type in sera of rheumatoid arthritis patients; correlations with anti-Hsp40 immune response and diagnostic markers. Acta Biochim Pol. 10.18388/abp.2010_2412 20827447

[38] Mantej J, Polasik K, Piotrowska E et al (2019) Autoantibodies to heat shock proteins 60, 70, and 90 in patients with rheumatoid arthritis. Cell Stress Chaperones 24:283–287. 10.1007/s12192-018-0951-9 PMC6363621 30465159

[39] Androvitsanea A, Stylianou K, Drosataki E et al (2021) The pathophysiological role of heat shock response in autoimmunity: a literature review. Cells 10:2626. 10.3390/cells10102626 PMC8533860 34685607

[40] Zhai Y, Wu K, Lin Q et al (2025) Post-Translational Modified Neoantigens in Autoimmune Diseases: Challenges of Immune Tolerance. Adv Sci 12:e01766. 10.1002/advs.202501766 PMC12442617 40538197

[41] Cooper GS, Dooley MA, Treadwell EL et al (1998) Hormonal, environmental, and infectious risk factors for developing systemic lupus erythematosus. Arthritis Rheum 41:1714–1724. 10.1002/1529-0131(199810)41:10<1714::aid-art3>3.0.co;2-u 9778212

[42] Horton DB, Strom BL, Putt ME et al (2016) Epidemiology of Clostridium difficile Infection–Associated Reactive Arthritis in Children: An Underdiagnosed, Potentially Morbid Condition. Jama Pediatr 170:e160217–e160217. 10.1001/jamapediatrics.2016.0217 PMC5069192 27182697

[43] Wheeler AM, Baker JF, Poole JA et al (2022) Genetic, social, and environmental risk factors in rheumatoid arthritis-associated interstitial lung disease. Semin Arthritis Rheum 57:152098. 10.1016/j.semarthrit.2022.152098 PMC10123759 36155967

[44] Wang S, Zhang X, Zhang Y et al (2025) The pathogenesis and therapeutic strategies of heat stroke-induced endothelial injury. Front Cell Dev Biol 13:1569346. 10.3389/fcell.2025.1569346 PMC12284237 40703654

[45] Walter EJ, Carraretto M (2016) The neurological and cognitive consequences of hyperthermia. Crit Care 20:199. 10.1186/s13054-016-1376-4 PMC4944502 27411704

[46] Briaud P, Frey A, Marino EC et al (2021) Temperature influences the composition and cytotoxicity of extracellular vesicles in *Staphylococcus aureus*. mSphere 6:10.1128/msphere.00676 − 21. 10.1128/msphere.00676-21 PMC8510519 34612674

[47] Bewicke-Copley F, Mulcahy LA, Jacobs LA et al (2017) Extracellular vesicles released following heat stress induce bystander effect in unstressed populations. J Extracell Vesicles 6:1340746. 10.1080/20013078.2017.1340746 PMC5505002 28717426

[48] Vomero M, Lamberti L, Corberi E et al (2025) Specialized pro-resolving mediators and autoimmunity: recent insights and future perspectives. Autoimmun Rev 24:103896. 10.1016/j.autrev.2025.103896 40744143

[49] Basil MC, Levy BD (2016) Specialized pro-resolving mediators: endogenous regulators of infection and inflammation. Nat Rev Immunol 16:51–67. 10.1038/nri.2015.4 PMC5242505 26688348

[50] McKinney EF, Smith KG (2016) T-cell exhaustion: understanding the interface of chronic viral and autoinflammatory diseases. Immunol Cell Biol 94:935–942. 10.1038/icb.2016.81 27577866

[51] McKinney EF, Lee JC, Jayne DRW et al (2015) T-cell exhaustion, co-stimulation and clinical outcome in autoimmunity and infection. Nature 523:612–616. 10.1038/nature14468 PMC4623162 26123020

[52] Chatpar PC, Muller D, Gruber B et al (1986) Prevalence of IgE rheumatoid factor (IgE RF) in mixed cryoglobulinemia and rheumatoid arthritis. Clin Exp Rheumatol 4:313–317 3791715

[53] Gorevic PD (2012) Rheumatoid Factor, Complement, and Mixed Cryoglobulinemia. J Immunol Res 2012:439018. 10.1155/2012/439018 PMC3432568 22956968

[54] Staal J, Blanco LP, Perl A (2023) Editorial: mitochondrial dysfunction in inflammation and autoimmunity. Front Immunol 14:1304315. 10.3389/fimmu.2023.1304315 PMC10582977 37860005

[55] Jing W, Liu C, Su C et al (2023) Role of reactive oxygen species and mitochondrial damage in rheumatoid arthritis and targeted drugs. Front Immunol 14:1107670. 10.3389/fimmu.2023.1107670 PMC9948260 36845127

[56] Yang Z, Shen Y, Oishi H et al (2016) Restoring oxidant signaling suppresses proarthritogenic T cell effector functions in rheumatoid arthritis. Sci Transl Med 8:331ra38. 10.1126/scitranslmed.aad7151 PMC5074090 27009267

[57] Doherty E, Oaks Z, Perl A (2014) Increased Mitochondrial Electron Transport Chain Activity at Complex I Is Regulated by N-Acetylcysteine in Lymphocytes of Patients with Systemic Lupus Erythematosus. Antioxid Redox Signal 21:56–65. 10.1089/ars.2013.5702 PMC4048573 24673154

[58] An J, Liu Y, Wang Y et al (2022) The role of intestinal mucosal barrier in autoimmune disease: a potential target. Front Immunol 13:871713. 10.3389/fimmu.2022.871713 PMC9284064 35844539

[59] Rio P, Caldarelli M, Gasbarrini A et al (2024) The Impact of Climate Change on Immunity and Gut Microbiota in the Development of Disease. Diseases 12:118. 10.3390/diseases12060118 PMC11202752 38920550

[60] Alirezaei M, Fox HS, Flynn CT et al (2009) Elevated ATG5 expression in autoimmune demyelination and multiple sclerosis. Autophagy 5:152–158. 10.4161/auto.5.2.7348 PMC2779564 19066443

[61] Kovacs JR, Li C, Yang Q et al (2012) Autophagy promotes T-cell survival through degradation of proteins of the cell death machinery. Cell Death Differ 19:144–152. 10.1038/cdd.2011.78 PMC3252822 21660048

[62] Yang Z, Fujii H, Mohan SV et al (2013) Phosphofructokinase deficiency impairs ATP generation, autophagy, and redox balance in rheumatoid arthritis T cells. J Exp Med 210:2119–2134. 10.1084/jem.20130252 PMC3782046 24043759

[63] McCormick JJ, King KE, Côté MD et al (2020) Regulation of autophagy following ex vivo heating in peripheral blood mononuclear cells from young adults. J Therm Biol 91:102643. 10.1016/j.jtherbio.2020.102643 32716884

[64] King KE, McCormick JJ, Kenny GP (2024) Temperature-Dependent Relationship of Autophagy and Apoptotic Signaling During Cold‐Water Immersion in Young and Older Males. Adv Biol 8:e2300560. 10.1002/adbi.202300560 38150671

[65] Dong S, Li D, Shi D (2024) Skin barrier-inflammatory pathway is a driver of the psoriasis-atopic dermatitis transition. Front Med 11:1335551. 10.3389/fmed.2024.1335551 PMC11007107 38606161

[66] Bellocchi C, Carandina A, Montinaro B et al (2022) The interplay between autonomic nervous system and inflammation across systemic autoimmune diseases. Int J Mol Sci 23:2449. 10.3390/ijms23052449 PMC8910153 35269591

[67] Wigley FM, Flavahan NA (2016) Raynaud’s Phenomenon. N Engl J Med 375:556–565. 10.1056/nejmra1507638 27509103

[68] Denton CP, Khanna D (2017) Systemic sclerosis. Lancet 390:1685–1699. 10.1016/s0140-6736(17)30933-9 28413064

[69] Nihtyanova SI, Brough GM, Black CM et al (2008) Clinical burden of digital vasculopathy in limited and diffuse cutaneous systemic sclerosis. Ann Rheum Dis 67:120–123. 10.1136/ard.2007.072686 17660220

[70] Taylor L, Hansen D, Morrisroe K, Fairley J, Calderone A, Oon S, Ross L, Stevens W, Ferdowsi N, Quinlivan A, Sahhar J, Ngian G, Apostolopoulos D, Host LV, Walker J, Tabesh M, Proudman S, Nikpour M (2025) Impact of season, environmental temperature, and humidity on Raynaud phenomenon in an Australian systemic sclerosis cohort. Arthritis Care Res 77:61–8. 10.1002/acr.25452 39420564

[71] Virgili-Gervais G, Matthews B, Nassar E-L et al (2024) The association of outdoor temperature and self-reported Raynaud’s phenomenon severity among people with systemic sclerosis: a Scleroderma Patient-centered Intervention Network Cohort study. Lancet Rheumatol 6:e684–e692. 10.1016/s2665-9913(24)00189-9 39216493

[72] Khouri C, Roustit M, Cracowski J-L (2020) Impact of global warming on Raynaud’s phenomenon: a modelling study. F1000Research 9:829. 10.12688/f1000research.24939.1 PMC8220350 34322228

[73] Wu Q, Xu Z, Dan Y-L et al (2021) Low ambient temperature increases hospital re-admissions for systemic lupus erythematosus in humid subtropical region: a time series study. Environ Sci Pollut Res 28:530–537. 10.1007/s11356-020-10488-7 32815013

[74] Szeto C-C, Mok H-Y, Chow K-M et al (2008) Climatic influence on the prevalence of noncutaneous disease flare in systemic lupus erythematosus in Hong Kong. J Rheumatol 35:1031–1037 18398942

[75] Pyrpasopoulou A, Chatzimichailidou S, Aslanidis S (2012) Vascular disease in systemic lupus erythematosus. Autoimmune Dis. 10.1155/2012/876456 PMC3432322 22957213

[76] Wong N, Ng V-Y, Ibrahim S et al (2014) Lupus – the cold, hard facts. Lupus 23:837–839. 10.1177/0961203314530485 24699313

[77] Frischer JM, Weigand SD, Guo Y et al (2015) Clinical and pathological insights into the dynamic nature of the white matter multiple sclerosis plaque. Ann Neurol 78:710–721. 10.1002/ana.24497 PMC4623970 26239536

[78] Spiljar M, Steinbach K, Rigo D et al (2021) Cold exposure protects from neuroinflammation through immunologic reprogramming. Cell Metab 33:2231-2246.e8. 10.1016/j.cmet.2021.10.002 PMC8570411 34687652

[79] Berntsson S-G, Reis J, Zjukovskaja C et al (2025) Climate change impacts the symptomology and healthcare of multiple sclerosis patients through fatigue and heat sensitivity - a systematic review. J Neurol Sci 474:123526. 10.1016/j.jns.2025.123526 40359743

[80] Frohman TC, Davis SL, Beh S et al (2013) Uhthoff’s phenomena in MS—clinical features and pathophysiology. Nat Rev Neurol 9:535–540. 10.1038/nrneurol.2013.98 23732530

[81] Ogawa G, Mochizuki H, Kanzaki M et al (2004) Seasonal variation of multiple sclerosis exacerbations in Japan. Neurol Sci 24:417–419. 10.1007/s10072-003-0200-5 14767690

[82] Leavitt VM, Sumowski JF, Chiaravalloti N et al (2012) Warmer outdoor temperature is associated with worse cognitive status in multiple sclerosis. Neurology 78:964–968. 10.1212/wnl.0b013e31824d5834 PMC3310313 22402861

[83] Kiyatkin EA, Sharma HS (2009) Permeability of the blood–brain barrier depends on brain temperature. Neuroscience 161:926–939. 10.1016/j.neuroscience.2009.04.004 PMC2694729 19362131

[84] Sorensen C, Hess J (2022) Treatment and Prevention of Heat-Related Illness. N Engl J Med 387:1404–1413. 10.1056/nejmcp2210623 36170473

[85] Zhang Z, Gao X, Liu S et al (2025) Global, regional, and national epidemiology of rheumatoid arthritis among people aged 20–54 years from 1990 to 2021. Sci Rep 15:10736. 10.1038/s41598-025-92150-1 PMC11953469 40155668

[86] Chaseling GK, Uchmanowicz I, Bäck M et al (2025) Heat extremes and cardiovascular diseases: a scientific statement of the Association of Cardiovascular Nursing & Allied Professions, Association for Acute Cardiovascular Care, European Association of Preventive Cardiology, Heart Failure Association, European Heart Rhythm Association of the ESC, the ESC Council on Hypertension, the ESC Council on Stroke, and the ESC Working Group on Cardiovascular Pharmacotherapy. Eur Hear J 46:2950–2958. 10.1093/eurheartj/ehaf326 40457965

[87] Valle A, Dellaripa PF, Feldman CH (2026) The Effects of Extreme Heat on Individuals with Rheumatic Conditions Equity Considerations. Rheum Dis Clin North Am 52:33–46. 10.1016/j.rdc.2025.08.005 41265946

[88] Mruthyunjaya P, Sondur S, Ahmed S et al The climate emergency for rheumatologists: where do we stand? Clin Rheumatol 2024:1–10. 10.1007/s10067-024-07284-w 39710809

[89] Quitmann C, Griesel S, Schwerdtle PN et al (2023) Climate-sensitive health counselling: a scoping review and conceptual framework. Lancet Planet Heal 7:e600–e610. 10.1016/s2542-5196(23)00107-9 37438001

